# Remote dosimetric auditing of clinical trials: The need for vendor specific models to convert images to dose

**DOI:** 10.1002/acm2.12521

**Published:** 2018-12-31

**Authors:** Narges Miri, Philip Vial, Peter B. Greer

**Affiliations:** ^1^ School of Mathematical and Physical Sciences University of Newcastle Callaghan NSW Australia; ^2^ Department of Medical Physics Liverpool and Macarthur Cancer Therapy Centres Sydney Australia; ^3^ Ingham Institute of Applied Medical Research Sydney Australia; ^4^ Institute of Medical Physics School of Physics University of Sydney Sydney Australia; ^5^ South Western Sydney Clinical School University of New South Wales Sydney Australia; ^6^ Calvary Mater Newcastle Hospital Newcastle New South Wales Australia

**Keywords:** Elekta/Varian linacs, IMRT pretreatment dose verification, remote radiotherapy auditing

## Abstract

**Introduction:**

A previous pilot study has demonstrated the feasibility of a novel image‐based approach for remote dosimetric auditing of clinical trials. The approach uses a model to convert in‐air acquired intensity modulated radiotherapy (IMRT) images to delivered dose inside a virtual phantom. The model was developed using images from an electronic portal imaging device (EPID) on a Varian linear accelerator. It was tuned using beam profiles and field size factors (FSFs) of a series of square fields measured in water tank. This work investigates the need for vendor specific conversion models for image‐based auditing. The EPID measured profile and FSF data for Varian (vendor 1) and Elekta (vendor 2) systems are compared along with the performance of the existing Varian model (VM) and a new Elekta model (EM) for a series of audit IMRT fields measured on vendor 2 systems.

**Materials and methods:**

The EPID measured beam profile and FSF data were studied for the two vendors to quantify and understand their relevant dosimetric differences. Then, an EM was developed converting EPID to dose in the virtual water phantom using a vendor 2 water tank data and images from corresponding EPID. The VM and EM were compared for predicting vendor 2 measured dose in water tank. Then, the performance of the new EM was compared to the VM for auditing of 54 IMRT fields from four vendor 2 facilities. Statistical significance of using vendor specific models was determined.

**Results:**

Observed dosimetry differences between the two vendors suggested developing an EM would be beneficial. The EM performed better than VM for vendor 2 square and IMRT fields. The IMRT audit gamma pass rates were (99.8 ± 0.5)%, (98.6 ± 2.3)% and (97.0 ± 3.0)% at respectively 3%/3 mm, 3%/2 mm and 2%/2 mm with improvements at most fields compared with using the VM. For the pilot audit, the difference between gamma results of the two vendors was reduced when using vendor specific models (VM:* P* < 0.0001, vendor specific models: *P* = 0.0025).

**Conclusion:**

A new model was derived to convert images from vendor 2 EPIDs to dose for remote auditing vendor 2 deliveries. Using vendor specific models is recommended to remotely audit systems from different vendors, however, the improvements found were not major.

## 1 INTRODUCTION

Quality assurance (QA) is an essential procedure to assess accuracy of relevant parameters in radiotherapy^1^ while an external audit is recommended to assess consistency of local QA and effectiveness of delivery and measurement systems.^2^ The importance of external audits is emphasized in radiotherapy clinical trials where a consistent accuracy is essential.^3^, ^4^, ^5^ Conventional audits are performed by site‐visits or postal methods, which can be expensive and/or labor intensive.^6^, ^7^, ^8^ Some virtual methods have been explored to reduce the audit cost using in‐house QA methods.^9^

Recently a novel approach was introduced to remotely assess intensity modulated radiotherapy (IMRT) deliveries using pre‐treatment images from electronic portal imaging devices (EPIDs). The method was known as the Virtual Epid Standard Phantom Audit (VESPA) and designed for dosimetric auditing of clinical trials at remote facilities. The VESPA utilized an in‐house software for analysis and provided a relatively consistent detection system for data acquisition.^10^ Participating facilities were provided with CT data sets of the virtual water phantoms and transferred prostate and head and neck IMRT treatment plans onto these to calculate dose in their local treatment planning system (TPS). They electronically sent their images and planned dose to the auditing site for assessment.

The in‐house software of the VESPA back‐projects in‐air acquired images from EPIDs into virtual water phantoms and converts the signals to dose at 10 cm depth within the phantoms.^11^, ^12^ The conversion is performed based on a model developed by King et al. at Calvary Mater Newcastle Hospital (CMNH). The software input includes a machine specific file, a beam model file and DICOM images and doses. The machine specific file refines the input and adapts it to each machine/delivery system using the facility calibration images. This file includes parameters defining central axis coordinate on the EPID and EPID‐linac sag correction. Another software input is the beam model file referred to here as the Varian model (VM). The VM is not adjusted for each facility. It has been developed using aS1000 EPID acquired images from a Varian linac deliveries (vendor 1) of series of square fields. The beam profiles and field size factors (FSFs) of the deliveries were also measured in water tank and used for the VM optimization. The VM has been extensively benchmarked and used for vendor 1 in‐house QA.

Six facilities took part in a pilot study of the remote based auditing method. Three of the facilities acquired data from Varian delivery and measurement systems (vendor 1) and three from Elekta (vendor 2).^13^ The pilot study used the VM for both vendors but applied primary vendor differences to the machine specific file. Differences in the detector size and resolution were applied; vendor 1: aS1000 EPIDs with 40 × 30 cm^2^ active area, that is, 1024 × 768 image resolution with 0.039 cm pixel resolution and, vendor 2: iViewGT EPIDs with 41 × 41 cm^2^ active area, that is, 1024 × 1024 image resolution with 0.040 cm pixel resolution.^14^ Moreover, prior to analysis, acquired images at 160 cm source to detector distance (SDD) from vendor 2 were resampled to 100 cm. The “.HIS” format images acquired from iViewGT EPIDs were also converted to DICOM in consistent with the software input requirement. In spite of the applied differences to each machine file, slightly lower gamma pass rates were observed in the auditing results from vendor 2. The vendor 2 systems also demonstrated a different field size response for reconstructed dose at the phantom isocentre compared with those from vendor 1. These all could be due to the differences of relevant dosimetry characteristics between the two vendors. Ignoring the differences can result in significant uncertainties in the audit outcome.^15^ Accordingly, this research studies relevant dosimetric variations between the two vendors and corresponding dose conversion models. Then, it investigates whether using vendor specific models could make the audit results independent from the vendors.

This research investigates differences of the beam profiles and FSFs, for the two vendors. The parameters are used in the development of the image to dose conversion model which in turn is applied for data analysis of the remote EPID based audit. This study develops a model (EM) to convert images from EPID to dose inside the virtual phantom for vendor 2 deliveries. Then, the EM performance is compared with the VM for measured water tank data from vendor 2 deliveries. The EM is used for remote auditing of 54 IMRT fields from four vendor 2 facilities. Statistical study of the auditing results determines whether a vendor specific model is required for auditing of each vendor. This work will facilitate implementation of this new and efficient auditing procedure using a remote EPID based dosimetry with improved sensitivity.

## 2 MATERIALS AND METHODS

### 2.A Dosimetry

A series of square field beams, 3 × 3, 4 × 4, 6 × 6, 10 × 10, 15 × 15, 20 × 20, and 25 × 25 cm^2^, were delivered by a vendor 1 and a vendor 2 linac and, in‐air images were acquired by respectively an aS1000 and iViewGT EPID. The profiles and FSFs were acquired from the image signals to evaluate the differences of relevant dosimetric parameters between the two vendors. Note, the profiles and FSFs were later used for modeling signal to dose. The profiles were obtained from the pixel data in the crossplane through the central axis. The profiles penumbras were defined to quantify the profile differences. The penumbra widths were defined as the distance between 80% and 20% of the maximum dose for each side of the profile relative to central axis. The FSFs were directly extracted from the mean pixel value of the central 11 × 11 pixels of the image signals and, the difference between FSFs of the vendors was quantified by percentage differences as D = (D_vendor1_ − D_vendor2_) × 100/D_vendor1_.

An intra‐vendor study was conducted on four vendor 2 facilities to evaluate variations of their parameters. The facilities were called C_1_, C_2_, C_3_ and C_4_. The percentage difference was calculated for each facility (PD_C2, C3, C4_ = S_C1_ − S_C2, C3, C4_) × 100/S_C1_, (S: Signal). Later, the C_1_ image data were used to develop a new model (EM) for vendor 2. The relative consistency for vendor 1 facilities has been reported elsewhere.^16^, ^17^

### 2.B Modeling

Following the method of King et al.,^11^ which was used to develop a vendor 1 model (VM), a vendor 2 model (EM) was developed to convert images to dose onto the virtual phantom. Images from an iViewGT EPID and a vendor 2 measured dose in water tank (WT) were acquired. The images were acquired in‐air from delivery of series of square field beams, 3 × 3, 4 × 4, 6 × 6, 10 × 10, 15 × 15, 20 × 20, and 25 × 25 cm^2^. The water tank data were measured at 10 cm depth and used to optimize the model parameters. The water tank data were acquired at 100 cm SDD using a small cylindrical ionization chamber of CC01 for small field sizes, that is, 3 × 3, 4 × 4, 6 × 6 cm^2^, and a CC13 for the large field sizes, that is, 10 × 10, 15 × 15, 20 × 20, and 25 × 25 cm^2^. All images were acquired at 160 cm SSD and resampled to 100 cm SSD using interpolation. The images were truncated at about 1 cm of the detector edge to avoid the edge artefacts. As the images were found noisier than those from aS1000 EPIDs, an adaptive “wiener2” filter in MATLAB was used to reduce the image noise and its impact on the model convolution function. The “wiener2” low pass filters the images that have been degraded by a constant power additive noise. It uses a pixel wise adaptive method based on statistics estimated from a local neighborhood of each pixel.^18^ An initial trial EM could not consistently predict the FSFs for the four facilities. After investigation, an averaged FSF from the TPSs of the four facilities was used as the reference FSF for modeling purposes, see Supporting information. The EM model accuracy was quantified via calculating discrepancy between the image and water tank dose for the profiles and FSFs

(1) $$\text{ST}=\sum {\left(\frac{\text{image dose}-\text{water tank dose}}{\text{nfields}}\right)}^{2}$$

where “nfield” was number of dose measurements/points. Furthermore, percentage differences were calculated for the EM dose compared with water tank measured dose (WT) via (PD_EM_ = D_WT_ − D_EM_) × 100/D_WT_, (D: dose). The EM performance was then compared with the VM performance for estimating a vendor 2 water tank dose (WT). The percentage difference was calculated for both cases (PD_EM, VM_ = D_WT_ − D_EM, VM_) × 100/D_WT_, (D: dose).

### 2.C Auditing

The EM was used to convert pre‐treatment images from IMRT deliveries, a post‐prostatectomy (PP) and a head and neck (HN) plan, to dose for four vendor 2 facilities. Details of these plans and the audit procedures are detailed elsewhere.^10^, ^13^ Each facility delivered (7–9) IMRT fields per patient plan. For each field, the converted EPID dose was compared to corresponding TPS dose. The comparisons were performed by an in‐house developed gamma function at three different criteria, 3%/3 mm, 3%/2 mm, and 2%/2 mm. The EM performance was compared with the VM performance for the IMRT audits at 1%/1 mm gamma criteria. Finally, a statistical study was conducted on the pilot audit including facilities from both vendors to compare performance of the vendor specific models and VM solely applied to all facilities.

## 3 RESULTS

### 3.A Dosimetry

**Figure 1:**
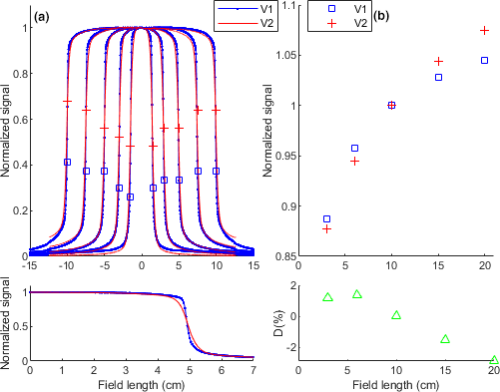
EPID measured signals for a vendor 1 and vendor 2 facility. (a) Beam profiles. Penumbras for V2 and V1 profiles were shown by respectively 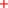 and 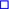. Note, penumbra unit is “cm”. The subplot magnifies the 10 × 10 cm^2^ profiles for comparison. (b) Field size factors (FSFs). The subplot demonstrates percentage differences for the FSFs. The profiles and FSF data were used to develop signal to dose conversion models (VM and EM).

Figure 1 demonstrates relevant parameters for the two vendors measured by corresponding EPIDs. As Fig. 1(a) demonstrates, the two vendors show some profile differences mainly in the horns and edge regions. Penumbras for vendor 2 and vendor 1 profiles were shown by respectively 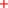 and .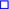 The penumbra values were demonstrated by the profile signal values but with a “cm” unit. For vendor 2, larger penumbras were observed at all field sizes. The Fig. 1(a) subplot magnifies the 10 × 10 cm^2^ profiles. It showed large differences in horn and edge of the profiles. As Fig. 1(b) demonstrates, FSFs of the vendor 2 are larger at large fields, >10 × 10 cm^2^, and smaller at small fields, <10 × 10 cm^2^, than other vendor. The percentage difference (D%) between FSFs of the vendors was better demonstrated in the subplot. The subplot shows largest discrepancy at the largest field sizes, that is, 20 × 20 cm^2^.

**Figure 2:**
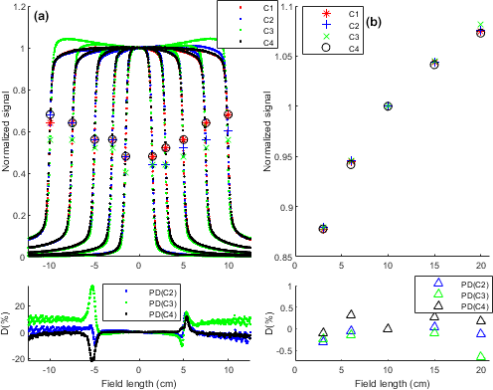
(a) EPID measured signals for four vendor 2 facilities. (a) Beam profiles. Penumbras for C_1_, C_2_, C_3,_ and C_4_ profiles were shown by respectively 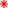, 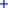, 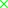 and ○. Note, penumbra unit is “cm”. The subplot demonstrates percentage differences for the 10 × 10 cm^2^ profiles. (b) Field size factors (FSFs) for the four facilities. The subplot shows percentage differences for the FSFs. The percentage difference was calculated by (PD_C2, C3,__C4_ = S_C__1_ − S_C__2, C3,__C4_) × 100/Sc_1,_ (S: Signal). Later, the C_1_ image data were used to develop a new model (EM) for vendor 2.

Figure 2 shows the signal response for four vendor 2 facilities measured by their iViewGT EPIDs. The signals were compared to the C_1_ values as the C_1_ was later used for the EM development. In addition to signal profiles, Fig. 2(a) shows values for the profiles penumbras. The penumbras were relatively similar for C_1_ and C_4_ and, for C_2_ and C_3_. However, a relatively large discrepancy was observed in penumbras of all facilities at the very large field, that is, 20 × 20 cm^2^. The subplot in Fig. 2(a) shows percentage difference for the 10 × 10 cm^2^ profiles. The largest difference was observed for C_3_ and the smallest for C_2_. Relatively similar trend was observed for other field sizes (not plotted). Figure 2(b) demonstrates the FSFs response for the four facilities and the subplot shows their percentage differences. For FSF, C_4_ shows a relatively large discrepancy at most fields and C_3_ shows the largest difference at the very large field, that is, 20 × 20 cm^2^.

### 3.B Modeling

**Figure 3:**
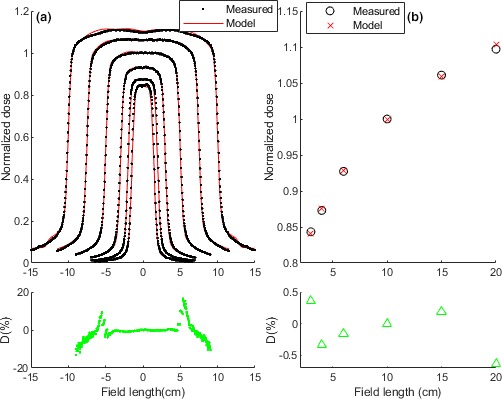
Measured dose by the new model (EM) compared with water tank measured data for a vendor 2 deliveries. (a) Dose profiles. The subplot shows percentage differences for the 10 × 10 cm^2^ profiles. (b) FSF dose. The subplot shows percentage differences for the FSFs. The percentage difference was calculated by (PD_EM_ = D_WT_ − D_EM_) × 100/D_WT_, (D: Dose).

Figure 3 demonstrates the EM estimated dose compared with water tank (WT) measured dose for a vendor 2 facility. The ST values for the profiles and FSFs were respectively 3.7 × 10^−6^ and 1.9 × 10^−6^ which were close to the values for the established VM, 2.1 × 10^−6^ and 1.53 × 10^−7^ respectively.^11^ The subplot of the Fig. 3(a) shows percentage difference of the dose profiles for the 10 × 10 cm^2^ profiles. The dips in the subplot came from the horns where the measured dose was smaller than the model dose. The peaks also originated from the profiles edge differences where the measured dose was larger than modeled dose. The dips/peaks demonstrated asymmetric response versus field size. Figure 3(b) shows the FSF dose measured by the EM and water tank (WT). The subplot showed the largest percentage difference at the very large field, that is, 20 × 20 cm^2^.

**Figure 4:**
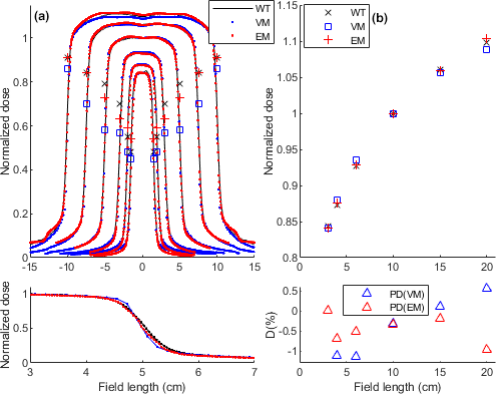
Performance of the two models (EM and VM) versus water tank (WT) dose for a vendor 2 deliveries. (a) Dose profiles. Penumbras for the EM, VM and WT profiles were shown by respectively 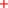, 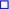 and ×. Note, penumbra unit is “cm”. The subplot magnifies the 10 × 10 cm^2^ profiles for comparison. (b) FSFs dose. The subplot shows percentage differences for the FSFs. The percentage difference was calculated by (PD_EM__,_ _VM_ = D_WT_ − D_EM__,_ _VM_) × 100/D_WT_, (D: dose).

Figure 4(a) compares a vendor 2 water tank (WT) dose profiles estimated by both models, that is, VM and EM. Penumbras for the EM, VM, and WT profiles were shown by respectively ,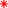 ,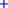 and ×. The EM penumbras were closer to the WT penumbras than the VM penumbras. The subplot magnifies the 10 × 10 cm^2^ profiles for a better visualization. A high agreement was observed between the EM and WT dose profiles. The Fig. 4(b) demonstrates the models calculated FSFs compared with the WT dose and the subplot shows percentage differences for the FSFs. Slightly better FSF estimation was observed for the EM than VM dose.

### 3.C Auditing

**Figure 5:**
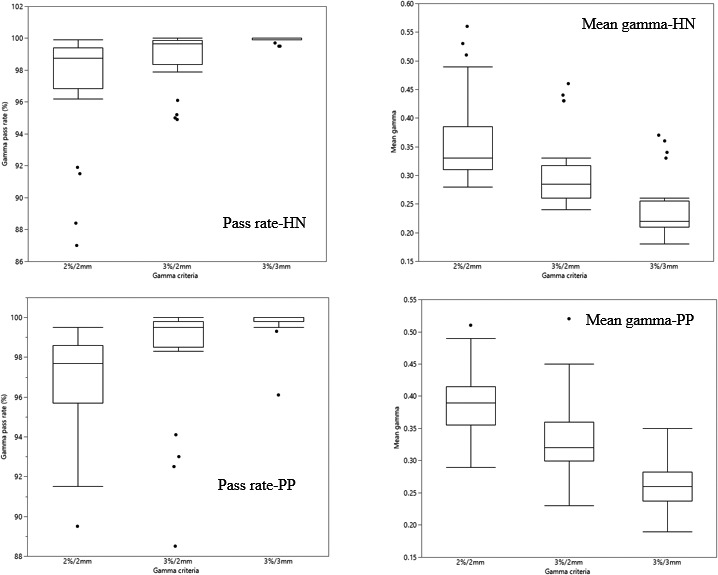
Auditing results of a post‐prostatectomy (PP) and a head and neck (HN) plan from four vendor 2 facilities, C_1_, C_2_, C_3,_ and C_4_, using the EM for analysis. Each facility has delivered (7–9) IMRT fields per treatment sites, totally 54 fields. The results include gamma pass rates and corresponding mean gammas for each patient plan.

**Figure 6:**
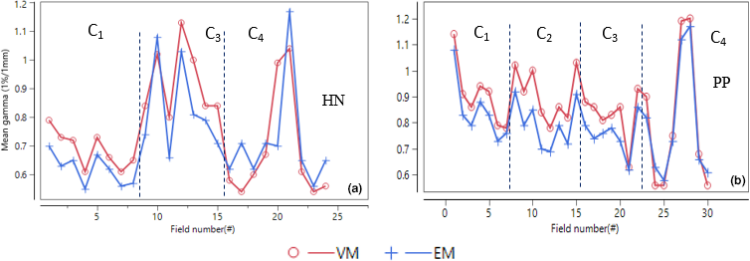
Mean gammas for the four vendor 2 centers for (a) head and neck (HN) and (b) Post‐prostatectomy (PP) patient plan using both the EM and VM.

Figure 5 summarizes the IMRT auditing results for vendor 2 facilities. The HN data from C_2_ were not considered in any analysis as they had acquired calibration images at a different date from other EPID measurements. The audit result of each treatment site was assessed by pass rate boxplots and corresponding mean gammas. The HN mean gamma pass rates were (99.9 ± 0.2)%, (98.8 ± 1.7)% and (97.1 ± 3.6)% at respectively 3%/3 mm, 3%/2 mm and 2%/2 mm. The mean pass rates for the PP were (99.8 ± 0.7)%, (98.4 ± 2.7)%, and (96.9 ± 2.5)% at the criteria. Interquartile ranges of the pass rates (mean gammas) at the gamma criteria were 0.1(0.05), 1.5(0.06), and 2.6(0.08) for the HN and 0.2(0.05), 1.3(0.06), and 2.9(0.06) for the PP. Figure 6 and Table 1 compare the auditing results for both the EM and VM using mean gamma values at 1%/1 mm criteria. Most of the HN and almost all PP fields from all facilities showed improved gamma results (lower mean gammas) for the EM than VM.

**Table 1:** Mean gamma pass rates at 1%/1 mm for four vendor 2 facilities and two patient plans using both the EM and VM

Centers	HN		PP	
	VM	EM	VM	EM
C_1_	78.5	83.5	66.1	69.4
C_2_	–	–	64.3	71.6
C_3_	63.1	69.3	68.5	73.1
C_4_	79.6	76.9	74.4	74.4

**Figure 7:**
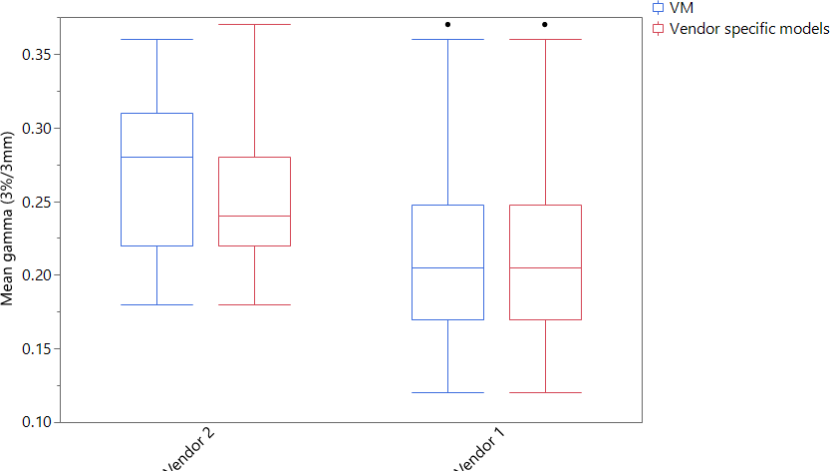
Auditing results for a study including two vendors. It uses either the VM or vendor specific models for dose conversion. The VM shows a significant difference between the two vendors (*P* < 0.0001). Using vendor specific models demonstrates less significant difference between the vendors (*P* = 0.0025).

Figure 7 compares results of the pilot audit when using the VM for both vendors (blue boxplots) and when using vendor specific models (red boxplots) at 3%/3 mm criteria. Using analysis of variance (ANOVA) and Tukey–Kramer HSD methods for comparison of the mean gammas for the two scenarios, the former demonstrated a significant audit difference between two vendors (*P* < 0.0001). The mean gamma difference for the two vendors was reduced when using vendor specific models (*P* = 0.0025).

## 4 DISCUSSION

The VESPA auditing procedure is designed as an inexpensive and efficient auditing procedure that can be performed remotely with the time for the central site physicist generally being 2–3 h to assess the results. The audit requires time from the local physicists to produce the IMRT verification plans and deliver the beams to the EPID, however, all other auditing methods require local personnel time. The VESPA also does not include any equipment or transport costs. The studies on the method has been conducted on two vendors using one vendor verified model (VM) to convert the image signal to dose inside the phantom. Investigation for the need for vendor specific models makes the audit reliable over different vendors.

Studies on relevant EPID measured dosimetric parameters showed differences between the two vendors. The discrepancy increased between the vendors’ profiles at the very small/large field sizes, ~3 × 3 and 20 × 20 cm^2^. The smaller penumbras observed for vendor 1 profiles indicate sharper profiles of corresponding images which may result in increasing the VM accuracy. The small penumbras for vendor 1 could be due to the proximity of the collimating system to the machine isocenter. For the FSFs of the two vendors, the discrepancy was increased by field size which was in accordance with the previous observations in the pilot audit. The FSF differences between the vendors could be due to differences in either EPID scatter or head scatter beam as the EPID signals incorporate both effects.

The study on vendor 2 facilities showed some inconsistencies in their dosimetric parameters. The C_3_ signals showed largest discrepancy with C_1_ signals at profiles, penumbras and FSFs. The C_2_ showed the minimum differences with the C_1_ profile but for penumbras and, the C_4_ showed the closest values to C_1_ penumbras. However, the FSF influence seems more important than the profiles impact for the model accuracy since the FSFs are used in optimizing four out of six model parameters while two parameters are tuned by profiles. A comparison between Figs. 1 and 2 shows larger inter‐vendor discrepancy (vendor 1 and vendor 2) than intra‐vendor variations (C_1_, C_2_, C_3_ and C_4_) for both parameters. This is in accordance with a report from Cozzi et al.^19^ and suggests developing a vendor 2 specific model may improve the auditing outcome.

A new model (EM) was developed for vendor 2 systems using a vendor 2 acquired parameters. The ST values for the EM were quite close to the values for the VM indicating high accuracy of the EM. Note, the VM has already been benchmarked and established as a reliable in‐house QA tool. The model calculated dose is compared to corresponding TPS dose. High sensitivity of the model to the planned discrepancies ensures that clinically significant dosimetric errors are detectable. An in‐house assessment demonstrated the method enough sensitivity to introduced MLC and/or collimator errors. However, a study on sensitivity of the gamma compared with a DVH approach is ongoing to determine the dose to the provided virtual patient CT dataset from the model. The model sensitivity to global dose differences is as expected dependent on the criteria with doses above the dose difference easily detected but those below it not.

The EM could accurately calculate water tank dose (WT) of a vendor 2 system. However, relatively large discrepancies were observed in horns and edges of the profiles. The EM dose also included small asymmetries in the profiles which may originate from the EPID image signals. Altogether, the EM was able to better calculate the WT dose profiles at all fields compare with the VM performance. For the FSFs, largest discrepancy of the EM with WT dose was observed at the very large field, that is, 20 × 20 cm^2^. For most of the fields, the EM slightly better estimated the FSFs than the VM did.

The auditing pass rates for the two IMRT plans were relatively high for all facilities at the three gamma criteria and, their corresponding mean gammas showed similar behavior. No significant difference was observed between the auditing results for the two treatment sites, the HN and PP. For the HN results, more outliers were observed in the gamma results than for the PP audits. This could be due to relatively lower number of auditing fields included for the HN studies. In addition to analysis by treatment site, the results were analysed for each facility. Except for C_4_, mean gammas for all facilities and treatment sites were smaller for the EM than the VM. For C_4_, the VM demonstrated relatively better response for the HN. The VM, moreover, showed relatively similar response to the EM for the PP. In general, using the EM for auditing vendor 2 facilities reduced mean gammas though, the differences between the EM and VM performances were not easily observed unless a highly strict gamma criteria, that is, 1%/1 mm, was used. This is in accordance with the above observations showing small improvement for calculating FSF dose.

The new EM and the VM were used to convert dose for deliveries from respectively vendor 2 and vendor 1 facilities in a study. The deliveries were also analysed using only VM for both vendors. Statistical studies of the two scenarios demonstrated a minor improvement when using vendor specific models (*P* = 0.0025) than the VM (*P* < 0.0001). Vendor dependency of the auditing results reduced when using vendor specific models (EM for vendor 2 and VM for vendor 1). However, mean gammas for vendor 2 were still larger than for vendor 1. This could be due to the impact of other variables such as facility TPS types which were not considered in this study.

## 5 CONCLUSION

Observed differences in relevant dosimetry parameters between vendor 1 and vendor 2 suggested using vendor specific models, to convert signal to dose onto the virtual phantoms, could account for dosimetry differences between the vendors. By developing a new model (EM) and using vendor specific models, the EM for vendor 2 and VM for vendor 1, the audit difference reduced between two vendors. The audit accuracy was improved and using vendor specific models was advised for future audits. The remote audit approach provides a highly automated method with significantly reduced cost.

## CONFLICT OF INTEREST

It is represented and warranted that, as at the date of this declaration, there is not any actual or perceived conflict of interest, or potential conflict of interest.

## Supporting information


**Fig. S1.** Gamma pass rates for both patients using both EM and VM. The VM shows better performance for most cases. (Each row represents results of each facility, C1, C2, C3, C4 respectively).
**Fig. S2.** Gamma pass rates for the VM and EM vs field size for the four facilities. The EM poor performance at fields ≤ 10 cm).
**Fig. S3.** The EM performance for different field sizes for the four facilities. Inconsistent response of the facilities.
**Fig. S4.** The images from iView images from the four facilities.
**Fig. S5.** Field size factors (FSFs calculated by TPSs of the facilities. The Clinac FSF is a TPS data used for the VM modeling.
