# Supplementary material for: Remote dosimetric auditing of clinical trials: The need for vendor specific models to convert images to dose
Source: J Appl Clin Med Phys. 2018 Dec 31;20(1):175–83. doi: 10.1002/acm2.12521 (PMC6333142; doi:10.1002/acm2.12521)
Supplement: Supplementary file 1 — Fig. S1. Gamma pass rates for both patients using both EM and VM. The VM shows better performance for most cases. (Each row represents results of each facility, C1, C2, C3, C4 respectively). Fig. S2. Gamma pass rates for the VM and EM vs field size for the four facilities. The EM poor performance at fields ≤ 10 cm). Fig. S3. The EM performance for different field sizes for the four facilities. Inconsistent response of the facilities. Fig. S4. The images from iView images from the four facilities. Fig. S5. Field size factors (FSFs calculated by TPSs of the facilities. The Clinac FSF is a TPS data used for the VM modeling. [file ACM2-20-175-s001.docx]

# Supplementary file

# Observation

- Initial EM was developed using water tank data (profiles & FSFs).

(b3, b4): are short term parameters trained using crossplane **profiles**

(b5-b8): are long term parameters trained using **FSFs**

(b1-b2): are Terma and Attenuation factors trained by **FSFs**

- When using EM for both the HN and PP patients for the 4 Elekta facilities, c1,c2,c3,c4:

The VM showed better performance than EM

For EM, the PP showed lower performance than H&N.

Main difference of patients: size.

Suggestion: The EM performance should be assessed for different field sizes.


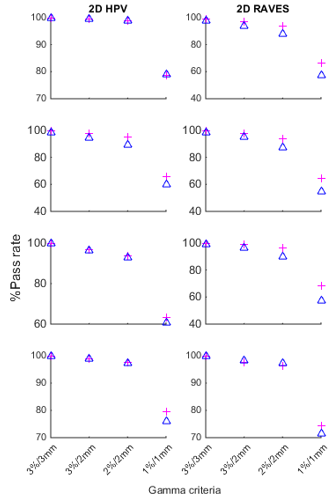


Figure 1- Gamma pass rates for both patients using both EM and VM. The VM shows better performance for most cases.(Each row represents results of each facility, C1,C2,C3,C4 respectively)

# Method

A- The performance of EM is compared with the VM performance for the FSFs of the facilities

B- Consistency of the EM performance is studied over different facilities

# Results


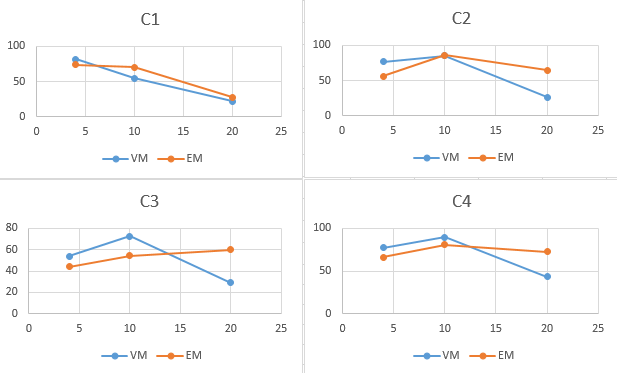


Figure 2- Gamma pass rates for the VM and EM vs field size for 4 facilities. The EM poor performance at fields<=10 cm)

Figure 3- The EM performance for different field sizes for the 4 facilities. Inconsistent response of the facilities.

- The EM poor performance at small fields
- Inconsistent EM response over the facilities

# Discussion

- A- The EM performance was good at large fields (>10cm) but poor at small fields. Could be from dosimetry inaccuracy at small fields (FSFs).

Suggestion: The water tank FSFs used for the EM optimisation could be replaced with averaged FSFs from TPS of the 4 facilities.

- B- For the EM, inconsistent gamma pass rates were observed between the facilities.

Gamma compares the EM with TPS and the comparison results shows the pass rates.

Then, the inconsistency could be from either the 1) EM or 2) TPS.

1- The EM investigation:

The EM was developed using the image data, profiles and FSFs, from one of the facilities.


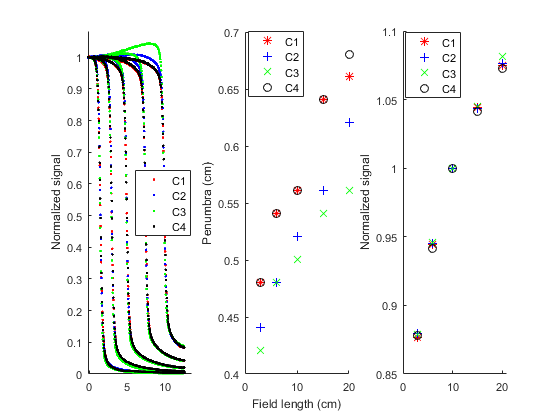


Figure 4- The images from iView images from 4 facilities.

The facilities images demonstrated small inconsistency in FSFs. The profiles however showed relatively large inconsistency.

Suggestion: Correct the profiles asymmetry manually. The suggestion was applied was no improvement was observed in the EM performance. These could be because 2 out of 6 parameters were optimized using profiles. The rest parameters were optimised using the FSFs. FSFs play more important role in model development while a small inconsistency was observed among FSFs. Then, the image data did not have a large impact on the EM development.

- The EM was trained/optimised using water tank data, profiles and FSFs,

Check for the accuracy of water tank data specially FSFs

Suggestion: replace the water tank FSFs with more accurate measurements

2) The TPS investigation: Calculated FSFs by the facilities’ TPS showed relatively large differences between FSFs of the different TPSs.

Suggestion: Average over calculated FSFs by different TPSs and use them to optimize the EM.


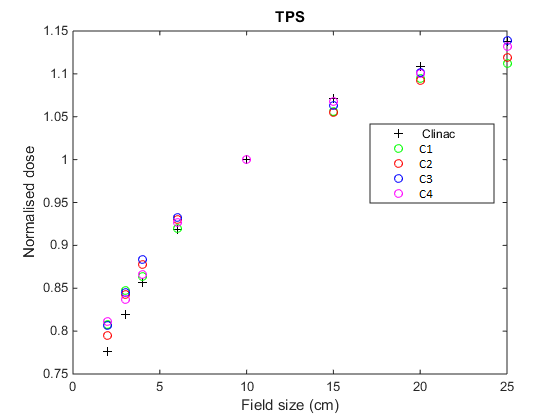


Figure 5- Field size factors (FSFs calculated by TPSs of the facilities. The Clinac FSF is a TPS data used for the VM modelling.

All above investigations resulted in training the EM by averaged FSFs of different TPSs calculations.
